# Novel Approach to Landfill Wastewater Treatment Fouling Mitigation: Air Gap Membrane Distillation with Tin Sulfide-Coated PTFE Membrane

**DOI:** 10.3390/membranes13050483

**Published:** 2023-04-29

**Authors:** Abdulaziz Khan, Ibrar Ibrar, Abeer Mirdad, Raed A. Al-Juboori, Priyamjeet Deka, Senthilmurugan Subbiah, Ali Altaee

**Affiliations:** 1 Mechanical and Mechatronic Engineering (MME), University of Technology Sydney, 15 Broadway, Sydney, NSW 2007, Australia; 2 Mechanical Department at Taif Technical College, Technical and Vocational Training Corporation (TVTC), Riyadh 11564, Saudi Arabia; 3 Centre for Green Technology, School of Civil and Environmental Engineering, University of Technology Sydney, 15 Broadway, Sydney, NSW 2007, Australia; 4 Faculty of Engineering and Information Technology, University of Technology Sydney, 5 Broadway, Sydney, NSW 2007, Australia; 5 NYUAD Water Research Centre, New York University-Abu Dhabi Campus, Abu Dhabi P.O. Box 129188, United Arab Emirates; raa9914@nyu.edu; 6 Department of Chemical Engineering, Indian Institute of Technology Guwahati, Guwahati 781039, India

**Keywords:** membrane distillation, high recovery, landfill leachate, fouling, membrane coating, tin sulfide coating

## Abstract

This study addressed the fouling issue in membrane distillation (M.D.) technology, a promising method for water purification and wastewater reclamation. To enhance the anti-fouling properties of the M.D. membrane, a tin sulfide (TS) coating onto polytetrafluoroethylene (PTFE) was proposed and evaluated with air gap membrane distillation (AGMD) using landfill leachate wastewater at high recovery rates (80% and 90%). The presence of TS on the membrane surface was confirmed using various techniques, such as Field Emission Scanning Electron Microscopy (FE-SEM), Fourier Transform Infrared Spectroscopy (FT-IR), Energy Dispersive Spectroscopy (EDS), contact angle measurement, and porosity analysis. The results indicated the TS-PTFE membrane exhibited better anti-fouling properties than the pristine PTFE membrane, and its fouling factors (FFs) were 10.4–13.1% compared to 14.4–16.5% for the PTFE membrane. The fouling was attributed to pore blockage and cake formation of carbonous and nitrogenous compounds. The study also found that physical cleaning with deionized (DI) water effectively restored the water flux, with more than 97% recovered for the TS-PTFE membrane. Additionally, the TS-PTFE membrane showed better water flux and product quality at 55 °C and excellent stability in maintaining the contact angle over time compared to the PTFE membrane.

## 1. Introduction

The negative environmental impact of leachate wastewater has been reported in the literature and has attracted researchers’ and scientists’ interest in the last decade [1,2]. Landfill leachate contains heavy metals, ammonia, organic pollutants, and other contaminants recognized by environmental organizations as hazardous compounds [3,4,5,6,7,8]. Membrane technologies have been proposed to treat leachate wastewater for reuse or safe discharge [9,10,11]. Nanofiltration (N.F.) and reverse osmosis (R.O.) are common pressure-driven membrane techniques used for wastewater and landfill leachate treatment [12,13,14]. Kristina and co-workers [15] used a two-stage R.O. membrane for landfill leachate treatment. The landfill leachate was prefiltered and pH was adjusted using the R.O. membrane, which achieved 43 L/m^2^ h water flux at the beginning of the test and then dropped to <15 L/m^2^ h after 90 h. The severe drop in water flux was attributed to membrane fouling by calcium compounds in the leachate wastewater. Weerapong et al. [16] studied short- and long-term R.O. membrane fouling in the treatment of landfill leachate. The short-term results showed insufficient cleaning with water, while chemical treatment with NaOH recovered > 90% of the initial water flux. In the long-term test, the maximum water recovery was no more than 35%. In another work, combined physicochemical and N.F. membrane processes were applied for landfill leachate treatment [17]. The results revealed that landfill leachate prefiltration was insufficient to prevent membrane fouling when the pressure was increased from 10 to 20 bar. Coagulation with FeCl_3_ resulted in a better water flux in the N.F. membrane, indicating insignificant irreversible fouling after coagulation. De Almeida R. et al. found that high capital and operating expenses also complicate the prevention of membrane fouling with R.O. and N.F. leachate treatment methods. Treating leachate with R.O. technology is projected to cost USD 8.58 per m³ [18].

Membrane distillation (M.D.) is an alternative technology suggested for leachate treatment that is less expensive to run and more resistant to fouling than N.F. and R.O. membranes [19]. Yan Z et al. [20] examined the AGMD system for leachate wastewater treatment. AGMD rejected approximately 99% of total organic carbon (TOC), phosphate, and metal ions. Inorganic scaling and organic fouling at a neutral feed pH lowered water flow, although fouling was alleviated by lowering the feed solution pH to the acidic range. In another study, silica oligomer fouling was reported in direct contact with M.D. (DCMD) at pH 5, while organic fouling was reported at pH 1 and pH 2. Therefore, feed acidification is not always beneficial in preventing M.D. fouling during leachate wastewater treatment [21]. Other researchers [22] investigated M.D. fouling during leachate treatment and concluded that foulants permeated the membrane pores when the conditions were alkaline. Complex fouling that included organic and inorganic components was found in alkaline conditions.

Membrane surface modification is a potential solution for alleviating fouling problems in M.D. technology. One of the most promising candidates in functionalization of the membrane surface is coating with graphene due to its integrated properties for a desired protective coating, such as unrestricted water permeation, superior hydrophilicity, bactericidal effects, chemical stability, and a strong and planar structure [23,24,25,26,27,28,29]. Recently, the use of graphene (G) substances has been proposed for coating or changing M.D. membranes to make them more resistant to scaling and fouling [30,31]. Researchers introduced the new concept of anti-fouling solar vapor gap membrane distillation (SVGMD) using a custom-designed multifunctional light absorber based on a graphene array for heating the water film transported through graphene nanochannels [32]. SVGMD exhibited an 82% water conversion ratio and excellent anti-fouling properties. Ren Jing et al. [21] suggested using the PTFE membrane’s graphene oxide (G.O.) coating for treating coking wastewater to improve M.D. water flux and fouling properties. The contact angle of the GO-PTFE membrane in air and water was 77.5° and 140.2°, respectively. The GO-PTFE membrane could let through 44% more water than the PTFE membrane, making it less likely to get blocked. A modified PVD with graphene (G-PVDF) was tested for treating coal seam gas wastewater using AGMD [33]. A small amount of graphene on the PVDF membrane produced superior water flux and higher rejection than a conventional PVDF membrane. Graphene was used to increase M.D. membranes’ water flux and fouling resistance. The graphene-coated PTFE membrane generally displayed improved water flux and fouling resistance due to its surface’s multiscale roughness [34,35,36]. Physical coating of the M.D. membrane is an intriguing technique for the chemically free incorporation of graphene on the membrane surface. A graphene dispersion was deposited on the PTFE membrane under 1 bar of pressure using a dead-end filtrated cell. As reported in [37], there is no need for chemical techniques since graphene nanosheets strongly adhere to polymer membranes via van der Waals interaction.

The adhesion energy between a single graphene layer and a silicon oxide substrate was measured to be 0.45 J/m^2^. Aljumaily and coworkers [38] fabricated superhydrophobic PVDF-HFP membranes for direct contact membrane distillation (DCMD) by incorporating powder-activated carbon (PAC). The results showed that the PAC was successfully coated onto the PVDF-HFP membrane, increasing contact angle values, porosity, and water distillate flux with each increment in PAC loading weight. The coated membranes with 30 mg PAC led to enhanced permeate flux with higher flux obtained at higher PAC loading, and they also exhibited an elevated salt rejection rate of more than 99.9% in most cases [38]. Chin et al. [39] found that enhancing the membrane’s surface superhydrophobicity is important for preventing membrane wetting. A P.P. membrane was coated using a solvent exchange method, which deposited additional polypropylene (P.P.) coating and enhanced the membrane’s superhydrophobicity. The coating produced uniform polymer spherulites on the membrane surface and increased the surface roughness of the membrane. A single-layered P.P. coating achieved a superhydrophobic surface with a high static water contact angle of 152.2°. Compared to uncoated membranes, the coated membrane also improved M.D. permeate flux by 30%, with an average of 13.0 kg/m^2^ h. The study found that the evenness of the surface coating and the size of the aggregate P.P. spherulites were significant factors that contributed to the membrane’s superhydrophobicity.

This study proposes the use of tin sulfide (SnS_2_) as a coating for PTFE membranes within the AGMD system used for treating landfill leachate. TS was used for the first time for PTFE membrane coating. It was selected in this study due to its hydrophobicity, thermal stability (melting point of 600 °C), and water insolubility. Instead of a chemical coating, a simple physical coating was applied. The TS-PTFE membrane was studied and applied for leachate reclamation. Membrane fouling was investigated at 80% to 90% recovery rates and 55 °C and 65 °C to assess the impact of feed temperatures on membrane fouling. As an environmentally friendly cleaning procedure, the TS-PTFE membrane was washed using DI hot water at 55 °C and 65 °C [40]. Several analytical techniques, including field emission scanning electron microscopy (FE-SEM), Energy Dispersive Spectroscopy (EDS), Fourier Transform Infrared Spectroscopy (FT-IR), contact angle (A.C.) measurement, and porosity analysis (P.A.), were used to examine the fouling layer on the membrane.

## 2. Materials and Methods

### 2.1. Feedwater Samples and Chemicals

The current study utilized wastewater from a landfill leachate treatment facility in Sydney, New South Wales, Australia, as the feed solution in a series of experiments. The landfill leachate was collected and cooled in a dark room to allow for the settlement of large particles before use. The properties of the collected landfill leachate are presented in Table 1.

The wastewater was analyzed using a combination of standard procedures and analytical instruments, such as Inductively Coupled Plasma Spectroscopy (ICP-MS) [41]. Tin sulfide with a purity level of 99.5% was provided by MSE Supplies located in Tucson, AZ, USA. Tin sulfide, also referred to as tin disulfide or stannic sulfide, is a valuable semiconductor material characterized by a band gap of 2.2 eV, n-type conductivity, and an average Seebeck coefficient ranging between 100 and 700 V/K [42,43].

### 2.2. PTFE Membrane Specifications

The PTFE membranes were supplied by Membrane Solutions, China. The membranes possess exceptional thermal stability and mechanical durability, making them heat resistant. The relevant properties of the PTFE membranes are detailed in Table 2. The active surface area of a PTFE membrane measures 0.0045 m^2^, which was placed on one side of the AGMD system, while the spacer frame was placed on the opposite side.

### 2.3. TS-PFTE Membrane Coating

In this study, the surface of a PTFE membrane was coated with SnS_2_ nanoparticles at room temperature. A small amount of TS nanopowder, weighing nearly 0.02 g, was placed on the surface of the PTFE membrane, covering an area of 0.0084 m^2^. The powder was evenly spread using a fingertip under a pressure of 1 bar. Any excess TS was removed from the TS-PTFE membrane surface using a clean and dry cotton cloth. A visualization is presented for the coating later. Depending on the experimental temperature, the TS-PTFE membrane was washed with DI water for 2 h at either 55 °C or 65 °C to remove loose TS particles and ensure a uniform coating on the membrane surface. The coating of the pristine PTFE membrane was confirmed with different analytical tools, such as SEM, FT-IR, and EDX.

### 2.4. AGMD Module Setup

**Figure 1:**
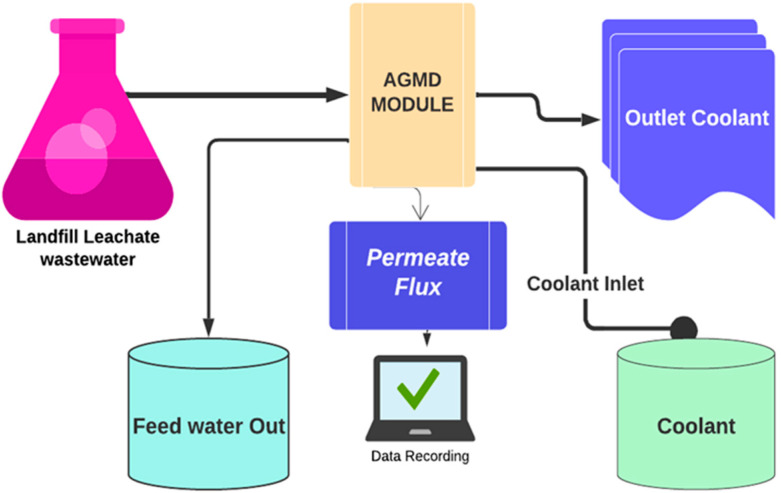
Schematic diagram of the AGMD system.

The AGMD crossflow module was designed with a transparent Plexiglas cell to facilitate visual inspection. A crossflow setup was used to prevent a concentration gradient. The module measures 130 mm in height, 180 mm in length, and 44 mm in depth and consist of three parts: cooling, feeding, and permeate collection. Eight bolts and nuts integrate these parts into a unified piece in the central section. The feed and cooling components have one exit point and one entrance point, with two hoses connecting the input and output. The permeating flux is facilitated through an outlet at the bottom of the AGMD setup. A spacer with dimensions of 70 (L) × 120 (W) × 0.2 (H) mm was inserted between the upper and lower components of the cell. The coolant side spacer frame had a total area of 0.0084 m^2^, while the feeding side spacer frame measured 0.0039 m^2^. The feed temperatures varied depending on the experiment’s objectives, with temperatures of 55 ± 3 °C or 65 ± 3 °C, while the coolant temperature was kept at roughly 18 ± 3 °C. The feed flow rate was 2.4 L/min, and the coolant solution flow rate was 1.6 L/min. The AGMD module setup is shown in Figure 1.

## 3. Experimental Methodology

The TS-PTFE membrane’s initial water flux and salt rejection were assessed at 55 °C and 65 °C using a feed solution of 35 g/L of sodium chloride (NaCl). The landfill leachate wastewater studies were conducted with input temperatures of 55 °C or 65 °C and recovery rates of 80% and 90%. In this study, the recovery percentage refers to the portion of water recovered from the feed after the treatment.

After the experiment, the TS-PTFE membrane was cleaned with DI water. Its water flux and rejection of a 35 g/L NaCl solution were re-evaluated and compared to the performance of the pristine membrane. The mathematical equation for calculating water flux (*WF*) is presented below:

(1) $$WF=\frac{V}{A*\Delta t}$$

Water flux (*WF*) is expressed in liters per square meter per hour (LMH) and is calculated using the permeate volume (*V* in liters), the active membrane area (*A* in m^2^), and the timescale (Δ*t* in h). After the feed treatment experiments, water flowing through the fouled TS-PTFE membrane was measured after it had been cleaned with deionized water at 55 °C or 65 °C for one hour. The fouling factor (*FF*) of the membrane after cleaning with deionized water was calculated at least twice using the following equation:

(2) $$FF=\frac{J_{i}-J_{a}}{J_{i}}*100$$

where $J_{i}$ represents deionized water flux before the treatment with landfill leachate wastewater and $J_{a}$ represents deionized water flux following the treatment with landfill leachate wastewater. The fouled TS-PTFE membrane was cleaned using deionized water at 55 °C or 65 °C for 60 min. A NaCl solution was used to evaluate the rejection of the TS-PTFE membrane after it had been cleaned with hot deionized water, and Equation (3) was used to calculate the rejection (Rj).

(3) $$Rj=\left(1-\frac{C_{p}}{C_{f}}\right)*100$$

where Rj is the rejection, $C_{p}$ is the quantity of salt in the permeate (mg/L), and $C_{f}$ is the quantity of salt in the feedwater (mg/L).

The feed and permeate samples’ rejection analyses were based on Na^+^ concentration measurements using ICP-MS analysis. Rejection was calculated using Equation (3). The same rejection for Cl^−^ ions was assumed based on the electroneutrality principle. The ICP-MS samples for all experiments were collected before and after filtration and then diluted according to the standards of ICP-MS. Once characterized, the dilution was converted back to the original values. The rejection of different ions was measured using Equation (3). The recovery rate (%*Re*) equation has been provided in the revised manuscript as the following:

(4) $$\;Recovery\;\left(\%Re\right)=\left(\frac{V_{p}}{V_{f}}\right)*100$$

*V_p_* is the permeate volume (L) and *V_f_* is the feed volume (L). The feed solution was heated to 55 °C or 65 °C and recycled in the AGMD system until the volume of recovered permeate reached 80% or 90% of the initial feed volume.

### 3.1. Membrane Characterizations by FE-SEM and EDX Analysis

FE-SEM and EDX were utilized to study fouling formation on the membrane surface. The FE-SEM examination used a Zeiss Supra 55 VP SEM equipped with a Schottky source and an acceleration voltage of 3 kV. EDX analysis of the pristine, cleaned, and fouled membranes was conducted using an Oxford detector. The membrane samples were dried at room temperature for 48 h in a clean room, with a gold coating then applied twice to the dried membranes. The double gold coating was used to enhance the conductivity of the specimen.

### 3.2. Pore Size, FT-IR Analysis, and Contact Angle Analysis

The Tech Inc Technology Techporo-AL-500 was employed to measure the pore diameters of the clean and fouled membranes. Fourier Transform Infrared Spectroscopy (FT-IR) was conducted using a Thermo Scientific Nicolet 6700 FT-IR spectrometer to examine the functional groups of both the clean and fouled membranes, with each scan being performed at least twice. The sessile drop technique and the CAM101 Contact Angle Analyzer were used to determine the contact angle by taking the average readings from various points on the membrane sample.

## 4. Results and Discussions

### 4.1. Impact of Temperature and Recovery Rate on TS-PTFE Membranes

**Figure 2:**
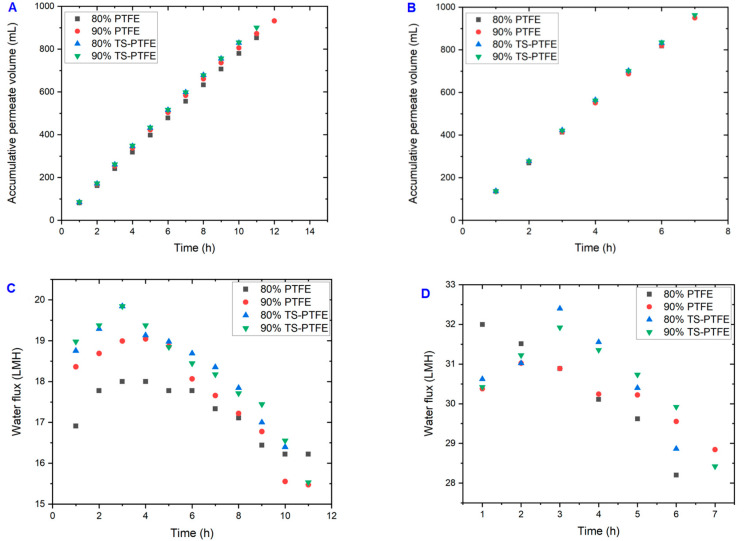
Cumulative permeate volume and water flux of PTFE and TS-PTFE membranes: (**A**) cumulative permeate volume of PTFE and TS-PTFE membranes at 55 °C; (**B**) cumulative permeate volume of PTFE and TS-PTFE membranes at 65 °C; (**C**) the water flux of PTFE and TS-PTFE membranes at 55 °C; (**D**) the water flux of PTFE and TS-PTFE membranes at 65 °C.

The performance of PTFE and TS-PTFE membranes was evaluated with landfill leachate wastewater at the two feed temperatures of 55 °C and 65 °C for 80% and 90% feed recovery rates. For the TS-PTFE membrane at 55 °C (Figure 2A), the total accumulated volume of permeate at 90% recovery was 901 ± 5 mL after 11 h, while it was 829 ± 5 mL in the 80% experiment after 10 h; the experimental time at 90% recovery was more than that at 80% recovery. It should be noted that the recovery rate would sometimes exceed 80% or 90% as the sample was collected hourly and the accumulated sample could reach the target for recovery before sample collection time elapsed. Nevertheless, the average permeate flow rate was 83.8 mL/h for the 80% recovery test and 82.5 mL/h for the 90% recovery test, indicating fairly steady membrane performance over time and recovery rates. Figure 2C shows that at 80% recovery, the water flux at 55 °C dropped by 17.34% from 19.84 LMH to 16.4 LMH. In contrast, water flux dropped by 22% from 19.9 LMH to 15.53 LMH during the 90% recovery test. The water flux drop was more significant at 90% recovery than at 80% recovery due to membrane fouling and wetting in the 90% recovery test, which required a longer time to reach. The TS-PTFE experiments also revealed that the average water flux at 90% recovery was only ~1.7% ± 0.2 lower than that at 80% recovery.

**Figure 3:**
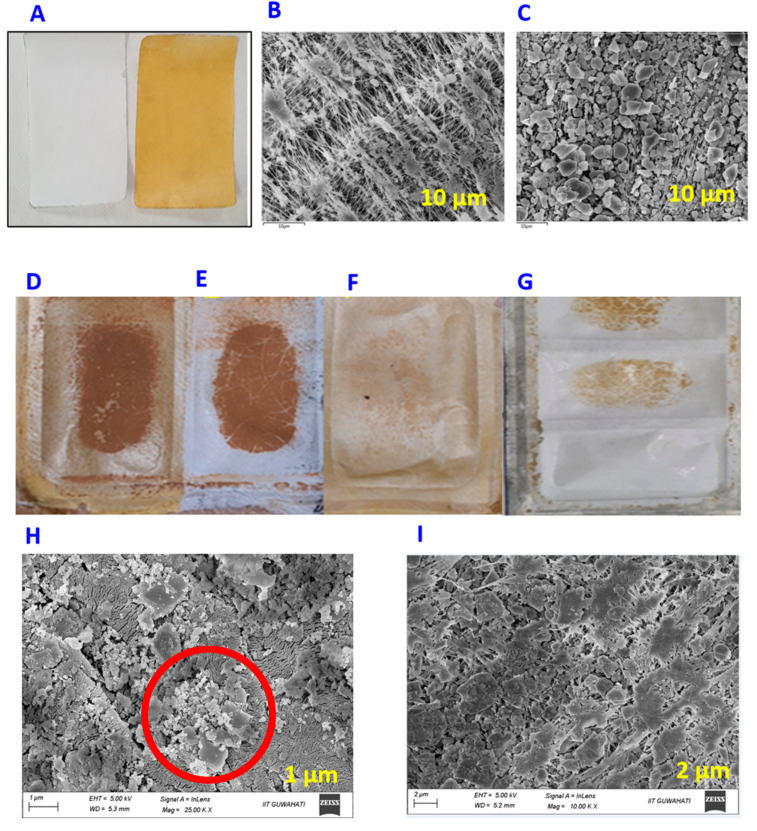
(**A**) Pristine PTFE and pristine TS-PTFE; (**B**) SEM image for pristine PTFE; (**C**) SEM image for TS-PTFE; (**D**) fouled TS-PTFE membrane before cleaning with hot DI water; (**E**) fouled PTFE membrane before cleaning; (**F**) fouled TS-PTFE membrane after cleaning with hot DI water; (**G**) fouled PTFE membrane after cleaning with hot DI water; (**H**) SEM image of the fouled PTFE membrane after cleaning with hot DI water showing foul material on the PTFE membrane surface; (**I**) SEM image of the fouled TS-PTFE membrane after cleaning with hot DI water.

Compared to the 55 °C tests, the average water flux increased by 39.6% for 80% recovery and 40.14% for 90% recovery at 65 °C (Figure 3D). The total accumulated volume of permeate reached 832 ± 5 mL and 963 ± 5 mL for the 80% and 90% recovery rates, respectively (Figure 2B). The higher feed temperatures increased feed vapor pressure, increasing water flux [44]. The smoothly decreasing water flux at the end of 80% and 90% recovery rate experiments is shown in Figure 2D. At 65 °C, the average permeate flow rate and the average water flux for the 90% recovery test were 135.35 mL/h and 30.57 LMH, with 136.21 mL/h and 30.81 LMH recorded for the 80% recovery test (Figure 2D). The water flux at 65 °C dropped by 10.8% from 32.4 LMH to 28.9 LMH during 80% recovery tests and by 11.3% from 32 LMH to 28.4 LMH during 90% recovery tests (Figure 2D). The relatively high water flux and long operation time at 65 °C feed temperature and 90% recovery rate promoted the movement of fouling materials to the membrane surface.

As the recovery rate increased from 80% to 90%, average water flux slightly decreased by 0.8%, showing steady membrane performance. Figure 2C,D show that increasing the feed temperature from 55 °C to 65 °C reduced the experimental time by 3 to 4 h, depending on the recovery rate, due to greater water flux at 65 °C. A previous study showed that water flow increased with feed temperature due to greater vapor pressure [45]. Generally, a tangible water flux increase was achieved by increasing the feed temperature from 55 °C to 65 °C. In contrast, average water flux was not significantly affected when the recovery rate increased from 80% to 90%, signifying steady membrane performance.

### 4.2. TS-PTFE Membrane Fouling and Cleaning

The landfill leachate wastewater was treated using membrane filtration at varying input temperatures and recovery rates with PTFE and TS-PTFE membranes. After completing the experiments, the fouled TS-PTFE membrane was cleaned using DI water for 60 min at either 55 °C or 65 °C, depending on the experimental temperature. It is noteworthy that the use of hot DI water was found to be an effective approach for removing fouling substances such as organic and inorganic particles that had accumulated on the membrane surface during the filtration process, as reported in previous studies [43]. As displayed in Table 1, leachate wastewater contains a combination of organic and inorganic pollutants that would foul the membrane. Pore blockage and membrane wetting are the primary factors for decreased water flux [46]. Scanning electron microscopy (SEM) was used to analyze pristine PTFE and TS-PTFE membranes before and after the filtration tests (Figure 3). Figure 3A shows the pristine PTFE membrane before and after coating with tin sulfide. Figure 3B,C display the SEM micrographs of uncoated PTFE membranes and those coated with tin sulfide. The yellowish coloration in Figure 3D–G is evidence of the accumulation of fouling materials on the surface of PTFE and TS-PTFE membranes before cleaning.

As shown in Table 3, the drop in the water contact angle of the TS-PTFE membrane, from 131.7° ± 3 to 85.6° ± 3 after 28 h of operation, was due to membrane fouling caused by metal hydroxide and organic matter deposition on the surface. The TS-PTFE membrane was cleaned at the end of each experiment via flushing with deionized water at either 55 °C or 65 °C for 60 min. The water flux, rejection rate, and fouling factor (FF) were then determined to evaluate the extent of the decrease in water flux and rejection caused by fouling of the TS-PTFE membrane.

**Figure 4:**
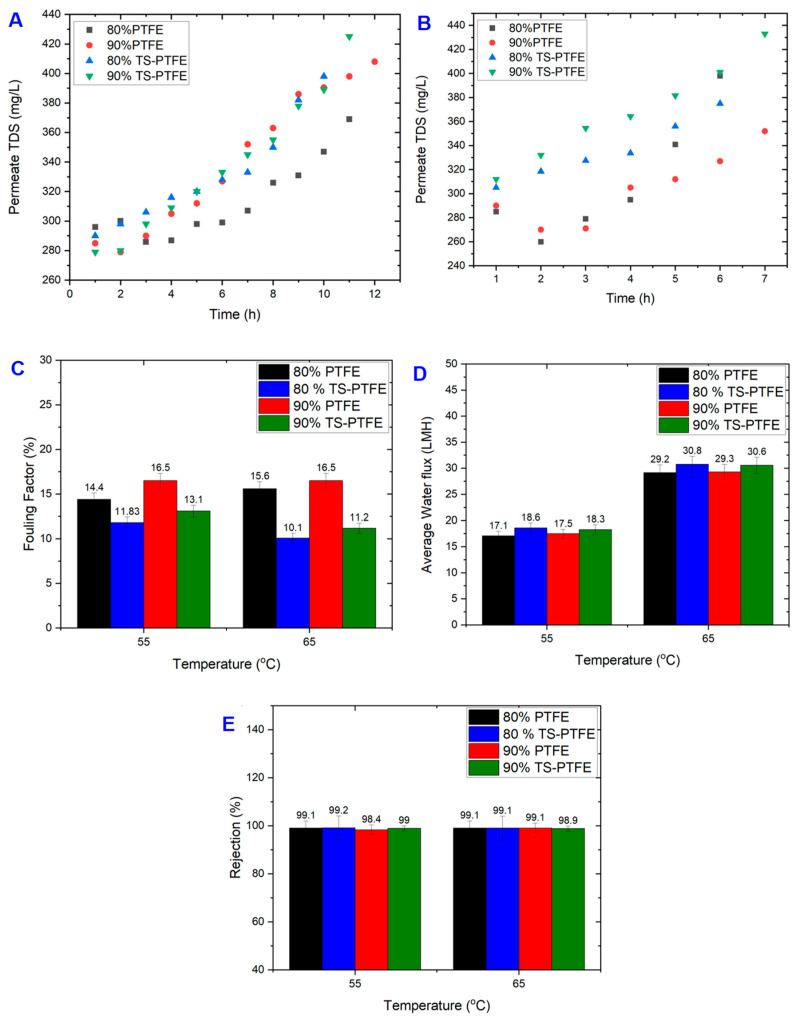
Permeate TDS for wastewater treatment with PTFE and TS-PTFE membranes. (**A**) Permeate TDS for wastewater treatment with PTFE and TS-PTFE membranes at 55 °C and 80% and 90% recovery; (**B**) permeate TDS for wastewater treatment with PTFE and TS-PTFE membranes at 65 °C and 80% and 90% recovery; (**C**) fouling factors after cleaning with DI water for PTFE and TS-PTFE membranes after 60 min of cleaning at 55 °C and 65 °C for all recoveries; (**D**) average water flux of PTFE and TS-PTFE membranes at 55 °C and 65 °C for all recoveries; (**E**) NaCl rejection for PTFE and TS-PTFE membranes for all recoveries at 55 °C and 65 °C. Rejection of NaCl was calculated for Na^+^ ions using ICP-MS analysis for feed and permeate samples (n = 3 for each test), and the average results are reported in (**E**).

Although membrane cleaning with hot DI water could effectively remove fouling substances, including organic and inorganic particles accumulated on the membrane surface during filtration [47], it is not effective in removing persistent fouling, which becomes thicker and more resistant over time as it builds up on the membrane surface (Figure 3F,H) [48]. Membrane analysis revealed decreases in the mean pore size of the TS-PTFE membrane of 11.5% and 11% after 20 h and 28 h, respectively, due to the deposition of fouling materials that resulted in membrane pore blockage, as indicated in Table 4. The range of fouling factors (FFs) after DI water cleaning for 80% and 90% recoveries at 55 °C was 11.8% and 13.11%, respectively, while the range at 65 °C was 10.2% and 11.3% (Figure 4C).

Figure 4A depicts permeate total dissolved solids (TDS) for the TS-PTFE membrane at 55 °C feed temperature. The TDS of the permeate increased by circa 30% for 80% and 90% recovery rates at a feed temperature of 55 °C. At 55 °C feed temperature, permeate TDS at 80% recovery exhibited a 27.4% increase (from 290 mg/L to 398 mg/L), while at 90% recovery they exhibited a 34.4% increase (from 279 mg/L to 425 mg/L) at the end of the experiments (Figure 4A). This increase is attributed to severe membrane fouling and wetting during the longer experimental time in the 90% recovery experiments, leading to a slight reduction in average water flux, as presented in Figure 4D.

The longer experimental time (11 h) in the 90% recovery test is believed to have contributed to membrane wetting and fouling, as indicated in FF, which went up by 9.7%, and average water flux, which dropped by 1.7%, as shown in Figure 4C,D. The FF in the 90% recovery experiments at 55 °C was around 10% greater than in the 80% recovery experiments at the same temperature. Generally, at 55 °C and 80% recovery, the fouling factor of the TS-PTFE membrane was 17.9%, which is less than that of the PTFE membrane at 55 °C and 90% recovery. At 65 °C and 80% recovery, the fouling factor of the TS-PTFE membrane was 34.6% lower than that of the PTFE membrane and 31.57% lower at 65 °C and 90% recovery. The percentage difference is substantial and was attributed to the anti-fouling properties of the TS-PTFE membrane.

In Figure 4B, TDS for the 80% and 90% recovery experiments at 65 °C feed temperature is presented. The results show an 18.7% increase in permeate TDS after 6 h for the 80% recovery test and a 27.9% increase in permeate TDS after 7 h for the 90% recovery test. The corresponding rise in concentrations was from 305 mg/L to 375 mg/L for the 80% recovery test and from 312 mg/L to 433 mg/L for the 90% recovery test. Thus, the NaCl rejection of pristine PTFE membranes is better than that of TS-PTFE membranes. The slight increase in the FF at 55 °C with the rise in feed temperature from 55 °C to 65 °C is attributed to the membrane fouling and wetting at 55 °C and the longer experimental time of 4 h [45], as shown in Figure 4C. It is worth noting that the experimental time at 65 °C was 3 to 4 h faster than at 55 °C feed temperature. The performance of the TS-PTFE membrane in terms of NaCl rejection post cleaning is depicted in Figure 4E. The rejection rate for experiments conducted at 55 °C ranged from 99.2% to 99%, and the rejection rate ranged from 99.1% to 98.9% for experiments performed at 65 °C. No significant differences in membrane rejection (about 0.2%) were observed for experiments conducted at 55 °C and 65 °C. The FF values for TS-PTFE membranes for all recoveries at 55 °C and 65 °C ranged from 10.2% to 13.1%, as illustrated in Figure 4C.

**Figure 5:**
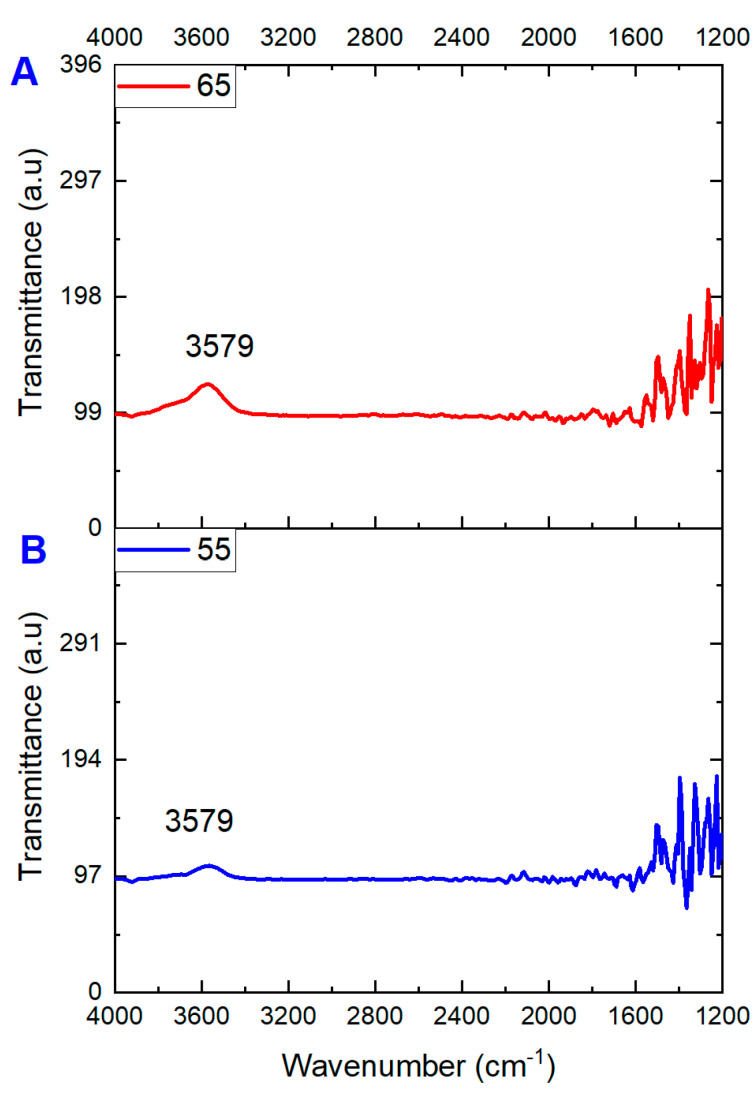
FT-IR spectra comparison of PTFE membranes fouled at (**A**) 65 °C and (**B**) 55 °C.

Figure 5 presents the FT-IR comparison of fouled PTFE membranes at 65 and 55 °C. The peak at 3579 cm^−1^ could potentially correspond to the O-H stretching of a hydroxyl group, a common functional group in many organic molecules. It can be attributed to the presence of humic substances in the landfill leachate wastewater (the yellowish color). This broad peak is often seen in the FT-IR spectra of organic compounds and is typically found in the range of 3200–3600 cm^−1^. This peak is more stretched for the higher temperature compared to the lower one in Figure 5B. Several other peaks can be seen from 1600 to 1200 cm^−1^, with several stretching and suppression peaks. The range of 1600 to 1200 cm^−1^ in an FT-IR spectrum can be attributed to the vibrations resulting from C=C and C=O bonds and can be mainly accredited to organic fouling in the landfill leachate wastewater. FT-IR analysis of fouled TS-PTFE membranes was also conducted to observe the differences between fouled membranes at 55 and 65 °C. The band at 3579 cm^−1^ (acetic acid group) shows slightly more stretching for 65 °C than the 55 °C spectrum. Overall, the spectrum appears visually similar, with more dense peaks for 65 °C from 1600 to 1200 cm^−1^.

### 4.3. Comparison of PTFE and TS-PTFE Performance

The performance of landfill leachate through PTFE and TS-PTFE membranes was evaluated under feed temperatures of 55 °C and 65 °C to achieve 80% and 90% recovery. It is well established that membrane fouling and wetting can negatively impact the filtration system’s water flux and rejection rate. Hot DI water cleaning was carried out to address this issue, which has been demonstrated as an effective method of removing fouling substances such as organic and inorganic particles loosely attached to the membrane surface that accumulate during the filtration process [47]. The fouling factor results shown in Figure 4C reveal that membrane cleaning with hot DI water was more effective in fouling alleviation for the TS-PTFE membrane than for the PTFE membrane. Nevertheless, some visible signs of fouling, characterized by a yellowish color, remained on the surface of the PTFE and TS-PTFE membranes after the 60 min hot DI water cleaning process, as shown in Figure 3F,H.

This study utilized scanning electron microscopy (SEM) and energy-dispersive X-ray spectroscopy (EDX) to examine the fouling layer on pristine and fouled PTFE and TS-PTFE membranes (Table 4). The SEM images show that the pristine membrane exhibited long fiber-like structures, as shown in Figure 3B. In contrast, these structures were barely visible in the fouled membranes, indicating the significant impact of fouling on membrane surface characteristics (Figure 3I,J). Furthermore, SEM analysis of the fouled membrane revealed a non-uniform layer of foulants along with a small crystalline salt structure on the surface, as indicated by nodule-like structures highlighted by a red circle in the center of the image in Figure 3H. The images in Figure 3F,H show a layer of fouling materials formed on the PTFE and TS-PTFE membrane surfaces after cleaning with hot DI water. The images show that the fouling layer on the PTFE membrane was thicker and more resistant to removal through cleaning with hot DI water than the layer on the TS-PTFE membrane.

In addition to SEM analysis, Table 3 shows the pore size and contact angle of pristine and fouled PTFE and TS-PTFE membranes. The contact angle of the PTFE membrane dropped by 20.9% and 27.9% after 15 and 30 h of operation, respectively. In contrast, the contact angle of the TS-PTFE membrane decreased by 33.2% and 34.9% after 20 h and 28 h of operation, respectively. However, the contact angle of the TS-PTFE membrane remained constant at about 86° ± 2 with an increase in operating time from 20 to 28 h; however, the contact angle of the PTFE membrane dropped by 8.8% from 102° to 93° with an increase in operating time from 15 to 30 h. Initially, the contact angle of the TS-PTFE membrane sharply dropped due to membrane fouling probably caused by high water flux, though it remained relatively stable over time. On the contrary, the drop in the contact angle of the PTFE membrane continued over time, indicating further membrane fouling. In the TS-PTFE membrane, the mean pore diameter decreased by 11.4% (from 0.175 μm to 0.154 μm) after 20 h of operation, as shown in Table 3. In comparison, the average pore diameter of the PTFE membrane decreased by 21.8% (from 0.248 μm to 0.194 μm) after 15 h of operation. The results indicate that the PTFE membrane underwent severe fouling, which resulted in a rapid contraction of the membrane’s pore diameter. However, operating the PTFE membrane for a more extended period, such as 30 h, caused an additional 12.4% reduction in pore size. The TS-PTFE membrane experienced no additional pore size contraction when operating time was extended to 28 h.

This study evaluated the average water flux of PTFE and TS-PTFE membranes at 55 °C and 65 °C under 80% and 90% recovery for landfill leachate treatment. Figure 4D shows that the average water flux of the PTFE membrane at 80% recovery reached 17.1 LMH at 55 °C and 29.2 LMH at 65 °C, while that of the TS-PTFE membrane reached 18.6 LMH at 55 °C and 30.8 LMH at 65 °C. The corresponding average water flux for 90% recovery was 17.5 LMH at 55 °C and 29.3 LMH at 65 °C for the PTFE membrane and 18.3 LMH at 55 °C and 30.6 LMH at 65 °C for the TS-PTFE membrane. The results in Figure 4D show that increasing the feed temperature by 10 °C resulted in an increase of 41.4% and 40.3% in the average water flux of the PTFE membrane at 80% and 90% recovery, while an increase of 39.6% and 40.14% in the average water flux of the TS-PTFE membrane at 80% and 90% recovery was observed with the same temperature increase [49]. The increase in water flux and the longer experiment time with higher recovery accelerates the transfer of fouling substances to the membrane surface, causing membrane fouling. The drop in contact angle from 129° to 93° in 30 h experiments for the PTFE membrane and from 131.4° to 85.6° in 28 h experiments for the TS-PTFE membrane at 55 °C (shown in Table 3) is attributed to membrane fouling, which renders the membrane surface more hydrophilic. The TS-PTFE membrane exhibited greater average water flux than the PTFE membrane due to its higher hydrophobicity, as shown in Table 3. Compared to the PTFE membrane, the TS-PTFE membrane exhibited an 8.7% and 4.6% increase in average water flux for 80% and 90% recovery rates at 55 °C and a 5.4% and 4.4% increase in average water flux for 80% and 90% recovery rates at 65 °C feed temperature (Figure 4D). A comparison between fouled and pristine PTFE and TS-PTFE membranes reveals that the PTFE membrane’s fouling was more severe than that of the TS-PTFE membrane, as depicted in Figure 3D–F,H and Figure 4C. Previous studies indicated that membrane fouling reduces permeate flux, which is potentially caused by pore blockage [49].

The significant drop in water flux exemplifies this observation in both PTFE and TS-PTFE experiments at 55 °C and 65 °C for all recovery rates (Figure 3D,E and Figure 4D), which is believed to be a result of membrane fouling. Outcomes indicate that average water flux was higher at 65 °C due to higher vapor pressure for both PTFE and TS-PTFE membranes. The increase in feed temperature from 55 °C to 65 °C resulted in a significant increase in average water flux, with the PTFE membrane experiencing growth from 17.1 to 29.3 LMH and the TS-PTFE membrane experiencing growth from 18.3 to 30.8 LMH. The results in Figure 4D show that increasing the feed temperature by 10 °C resulted in an increase of 41.4% and 40.3% in the average water flux of the PTFE membrane for both recovery rates, while an increase of 39.6% and 40.14% in average water flux was observed in the TS-PTFE membrane for both recovery rates [49].

The data presented in Figure 4C show the fouling factor of the PTFE and TS-PTFE membranes after cleaning with hot DI water. The fouling factor of the TS-PTFE membrane is generally lower than that of PTFE membranes at 55 °C and 65 °C due to the anti-fouling properties of the tin sulfide coating layer [50]. Figure 4C shows that at 55 °C feed temperature, the TS-PTFE membrane’s fouling factor was 17.8% to 20.5% lower than that of the PTFE membrane when operating at 80% and 90% recovery rates. At 65 °C feed temperature, the TS-PTFE membrane’s fouling factor was 34.6% to 29% lower than the PTFE membrane when operating at 80% and 90% recovery rates. The fouling factor for the TS-PTFE membrane was lower than that for the PTFE membrane due to its resistance to fouling and it probably being more responsive to cleaning with hot DI water. The fouling factor of the PTFE membrane was between 14.4% and 16.5% at 55 °C and between 15.6% and 16.5% at 65 °C feed temperature, while for the TS-PTFE membrane, it was between 11.83% and 13.11% at 55 °C and 10.2% and 11.29% at 65 °C feed temperature. These results suggest that cleaning with hot DI water is more effective for the TS-PTFE membrane than non-coated PTFE membranes when treating landfill leachate wastewater, as evidenced by all recoveries at both temperatures. Additionally, the results in Figure 4D display that the TS-PTFE membrane experienced an increase in average water flux at 55 °C of 8.1% at 80% recovery and 4.4% at 90% recovery, while at 65 °C average water flux was increased by 5.2% at 80% recovery and 4.2% at 90% recovery compared to PTFE at the same temperature.

Permeate total dissolved solids (TDS) for the TS-PTFE and PTFE experiments are shown in Figure 4. For the TS-PTFE experiment conducted at 55 °C feed temperature, permeate TDS increased by 27.4% and 34.4% during the experiments conducted at 80% and 90% recovery (Figure 4A). In contrast, at 65 °C feed temperature, permeate TDS increased by 23.2% and 28% during the experiments at 80% and 90% recovery (Figure 4B). The NaCl rejection of both PTFE and TS-PTFE membranes ranged from 98.4% to 99.2% at 55 °C and from 98.9% to 99.1% at 65 °C according to the results in Figure 4E. There was no significant difference in membrane NaCl rejection between the PTFE and TS-PTFE membranes at 55 °C feed temperature. For example, a difference of 0.7% was noted in the NaCl rejection of PTFE membranes between the 80% and 90% recovery experiments. This variation can be attributed to the longer experimental time (around one hour longer), which was required to achieve 90% recovery with a high amount of TDS compared to 80% recovery, resulting in membrane wetting.

The EDX results in Table 4 indicate a trace amount of elemental compositions covering the surface of pristine and fouled PTFE and TS-PTFE membranes after landfill leachate treatment. The carbon (C), magnesium (Mg), potassium (K), sodium (Na), and iron (Fe) ions in the fouling layer on the PTFE and TS-PTFE surfaces indicate the presence of organic and inorganic fouling. The sulfur (S) and tin (Sn) ions detected on the TS-PTFE membrane were from the tin sulfide coating layer. Organic–inorganic fouling mechanisms often involve organic molecules adhering to the hydrophobic membrane surface and divalent ions acting as bridging components [51]. The SEM analysis in Figure 3I,J shows that after 30 h and 28 h of operations, a dense layer of fouling material had formed on both the PTFE and TS-PTFE membrane surfaces due to membrane fouling.

The SEM images demonstrate that the PTFE membrane’s fouling became more noticeable and denser than on the TS-PTFE membrane. The analysis in Table 4 implies that organic and inorganic materials in the fouling layer were accountable for the PTFE and TS-PTFE membranes’ fouling [52]. The TS-PTFE membrane’s lower fouling factor was probably caused by the anti-fouling properties of the tin sulfide, which have been demonstrated in the literature [53]. The anti-fouling properties of tin sulfide hindered the fouling of the TS-PTFE membrane. Additionally, the TS-PTFE membrane’s fouling factor at 65 °C feed temperature was lower than that at 55 °C for both recovery rates, possibly due to the longer experimental time at 55 °C feed temperature.

## 5. Conclusions

In this study, for the first time, the PTFE membrane was coated with tin sulfide (SnS_2_) through a simple process that involved spreading the TS material on the PTFE membrane surface without the use of any chemicals. The effect of temperature on performance was studied in the AGMD unit, which was utilized to treat natural landfill leachate wastewater using PTFE and TS-PTFE membranes at feedwater temperatures of 55 °C and 65 °C to achieve high recovery rates. DI water was used to clean the PTFE and TS-PTFE membrane surfaces and remove fouling. The physical coating method proved robust even after 28 h of operation. The TS-PTFE membrane demonstrated improved performance and anti-fouling properties compared to the PTFE membrane, which is attributed to the anti-fouling properties of the TS nanoparticles. The fabricated TS-PTFE membrane was tested for up to 30 h with leachate wastewater to evaluate its water flux, NaCl rejection, and anti-fouling properties at 80% and 90% recovery rates. These testing conditions are challenging for the AGMD process since most previous studies considered lower recovery rates. Of note, membrane cleaning with DI water alone confirmed the excellent anti-fouling properties of the TS-PTFE membrane.

The results indicated a significant reduction in water flux in the PTFE membrane when temperature was decreased from 65 °C to 55 °C, with decreases of 41.4% and 40.3% for 80% and 90% recovery, respectively. The same decrease was observed for TS-PTFE, with a reduction of 39.6% and 40.14% for 80% and 90% recovery, respectively. The fouling factor of the PTFE membrane was approximately 20% higher than that of the TS-PTFE membrane at 55 °C and around 30% higher at 65 °C. The scanning electron microscopy (SEM) analysis showed that the surface of the TS-PTFE membrane was more resistant to fouling compared to the surface of the PTFE membrane.

The water contact angle analysis indicated that the hydrophobicity of the PTFE membrane changed continuously over time due to fouling. In contrast, the contact angle of the TS-PTFE membrane fell by 33.2% from 131.4° to 87.8° after 20 h and only decreased by a further 2.5% after 28 h of operation, remaining around 85.6°. An increase in feed temperature from 55 °C to 65 °C improved water flux for both PTFE and TS-PTFE membranes and shortened the operating time by 3–4 h for both recovery rates.

Cleaning the membrane with hot DI water was proven to be effective in restoring membrane performance. The FF reduction for the TS-PTFE membrane was lower than that of the pristine PTFE membrane, confirming the anti-fouling properties bestowed upon the membrane with the addition of TS. Future research should evaluate its feasibility in treating other saline feed solutions and different types of landfill leachate wastewater, as leachate composition can vary from one site to another. Tuning and optimizing the coating process is another potential future research avenue that merits exploration.

## Figures and Tables

**Table 1:** Characterizations of landfill leachate wastewater.

Parameter	Concentration	Measuring Instrument
Color	Brown yellowish	-
pH	8.01	HQ40d multi
Turbidity, NTU	35	2100P Turbidimeter
Conductivity, ms/cm	12.10	HQ14d Conductivity
TDS, mg/L	4500	
Total organic carbon, mg/L	145.1 ± 5	TOC analyzer (Shimadzu Corporation, Japan)
TSS, mg/L	27–117	7900 ICP-MS
Total irons, mg/L	3.5–52	7900 ICP-MS
Ammonia, mg/L	<0.5	5051—Ammonium Flow Plus ISE
Ca2+, mg/L	126 ± 5	7900 ICP-MS
Mg2+, mg/L	95.3 ± 5	7900 ICP-MS
K+, mg/L	47.87	7900 ICP-MS

**Table 2:** Membrane specifications.

Membrane Type	PTFE
Wettability	Hydrophobic
Nominal pore size, µm	0.45
Thickness, µm	146–223
Bubble point, psi	11.60–13.05
Flow rate, mL/min/cm^2^	43–52
Contact angle	129 ± 3°
TS-PTFE	131.6 ± 3°

**Table 3:** Pore size and contact angle of pristine and fouled membranes.

Membrane Type	Smallest Pore Diameter (µm)	Largest Pore Diameter (µm)	Mean Pore Diameter (µm)	Water Contact Angle (°)
PTFE pristine	0.213 ± 0.01	0.296 ± 0.01	0.248 ± 0.01	129 ± 2
TS-PTFE pristine	0.167 ± 0.01	0.248 ± 0.01	0.174 ± 0.01	131.4 ± 3
PTFE fouled 15 h	0.182 ± 0.08	0.296 ± 0.07	0.194 ± 0.07	102 ± 3
PTFE fouled 30 h	0.166 ± 0.06	0.244 ± 0.07	0.170 ± 0.08	93 ± 3
TS-PTFE fouled 20 h	0.151 ± 0.01	0.213 ± 0.01	0.154 ± 0.01	87.8 ± 3
TS-PTFE fouled 28 h	0.11 ± 0.01	0.248 ± 0.01	0.155 ± 0.01	85.6 ± 3

**Table 4:** Elemental composition (wt%) of pristine membranes and membranes fouled by landfill leachate.

Element	Pristine PTFE	Pristine TS-PTFE	TS-PTFE 20 h Fouled	TS-PTFE 28 h Fouled	PTFE 15 h Fouled	PTFE 30 h Fouled
C	56	71.9	66.3	71.6	70.42	44.01
Mg	0.3	0.3	0.9	0.62	0.90	1.42
Cl	0.60	-	-	-	0.19	0.29
K	0	-	0.04	-	0.20	0.29
Ca	0.1	0.041	0.035	0.03	0.20	0.39
Fe	1.7	0.03	0.034	0.07	0.66	2.06
S	-	4.2	5.3	4.1	-	-
Sn	-	8.2	9.2	8.2	-	-
Na	-	0	0.91	0.7	-	-
O	-	15.6	17.2	14.7	-	-

## Data Availability

The data presented in this study are available in the article.
